# Cytosolic TMEM88 promotes triple-negative breast cancer by interacting with Dvl

**DOI:** 10.18632/oncotarget.4379

**Published:** 2015-06-29

**Authors:** Xinmiao Yu, Xiupeng Zhang, Yong Zhang, Guiyang Jiang, Xiaoyun Mao, Feng Jin

**Affiliations:** ^1^ Department of Surgical Oncology and Breast Surgery, First Affiliated Hospital of China Medical University, Shenyang, China; ^2^ Department of Pathology, First Affiliated Hospital and College of Basic Medical Sciences, China Medical University, Shenyang, China

**Keywords:** cytosolic, TMEM88, triple-negative breast cancer, invasion, snail

## Abstract

TMEM88, a newly discovered protein localized on the cell membrane, inhibits canonical Wnt signaling. Immunohistochemic alanalysis of 139 breast cancers pecimens(64 triple-negative cancers and 75 non-triple-negative cancers) indicated that TMEM88 is expressed at significantly higher levels in breast cancer tissues (71.22%, 99/139) than in normal breast tissues (11.4%, 4/35; *p* < 0.001). The cytosolic and nuclear expression rates of TMEM88 were 57.81% and 9.37% in triple-negative and 52% and 33.33% (*p* = 0.5 and *p* = 0.001) in the non-triple-negative breast cancer tissues, respectively. Western blot analyses indicated that TMEM88 promoted Snail expression and inhibited Zo-1 and Occludin expression by interacting with dishevelled (Dvl) proteins, thereby stimulating invasion and metastasis in breast cancer. While cytosolic TMEM88 did not affect canonical Wnt signaling, cytosolic localization of this protein was positively correlated with both advanced TNM stage (*p* = 0.038 and *p* < 0.001) and lymph node metastasis (*p* = 0.01 and *p* = 0.002) in all and triple-negative specimens, respectively, and stimulated cell invasion by interacting with Dvls. Meanwhile, nuclear localization of TMEM88 was negatively correlated with lymph node metastasis (*p* = 0.046). Lastly, the increased prevalence of TMEM88 nuclear localization observed in non-triple-negative, compared to triple-negative tissues, suggests that the biological roles of TMEM88 differ depending on the subcellular localization.

## INTRODUCTION

While breast cancer survival rates have improved significantly over the last few decades, there is still no effective treatment for triple-negative breast cancer (ER-, PR-, and Her2- negative, TNBC) [1]. It is therefore essential to identify new biomarkers that can be used to predict tumor progression and that comprise potential therapeutic targets [2–7].

In a previous study, Lee et al. (2010) demonstrated that the C-terminal VWV (Val-Trp-Val) sequence of target protein transmembrane 88 (TMEM88), a potential two-transmembrane-type protein, interacts with the PDZ domain of dishevelled-1 (Dvl-1) in *Xenopus* embryos. Indeed, the results of this study indicated that TMEM88 might inhibit the canonical Wnt/β-catenin signaling pathway by competing with LRP5/6 for interaction with Dvl-1 [8]. Meanwhile, Palpant et al. (2013) showed that there are two TMEM88 isoforms: CRA-a(17 kDa), which inhibits the canonical Wnt/β-catenin pathway through interactions with Dvl proteins and regulates the development of myocardial cells, and CRA-b (25 kDa), which lacks the VWV motif and therefore likely does not interact with Dvl proteins [9].

While Lee et al. (2010) partially characterized the expression, subcellular location, and possible mechanisms of action of TMEM88 in *Xenopus* embryos [8], the expression pattern of this protein in human cells, particularly in malignant tumor cells, has yet to be investigated. Furthermore, it remains unclear whether the biological role of TMEM88 in tumor cells is dependent on its interaction with Dvl proteins. In this study, we examined the expression and subcellular localization pattern of TMEM88 in tissues obtained from 64 triple-negative breast cancer and 75 non-triple-negative breast cancer (ER-, PR-, and Her2- positive expression; hereafter referred to as triple-positive breast cancer, TPBC) patients. Furthermore, by modulating the expression levels of TMEM88, we assessed the role of this protein in the proliferation and metastasis of breast cancer cells.

## RESULTS

### Expression and distribution of TMEM88 in breast cancer specimens

**Figure 1:**
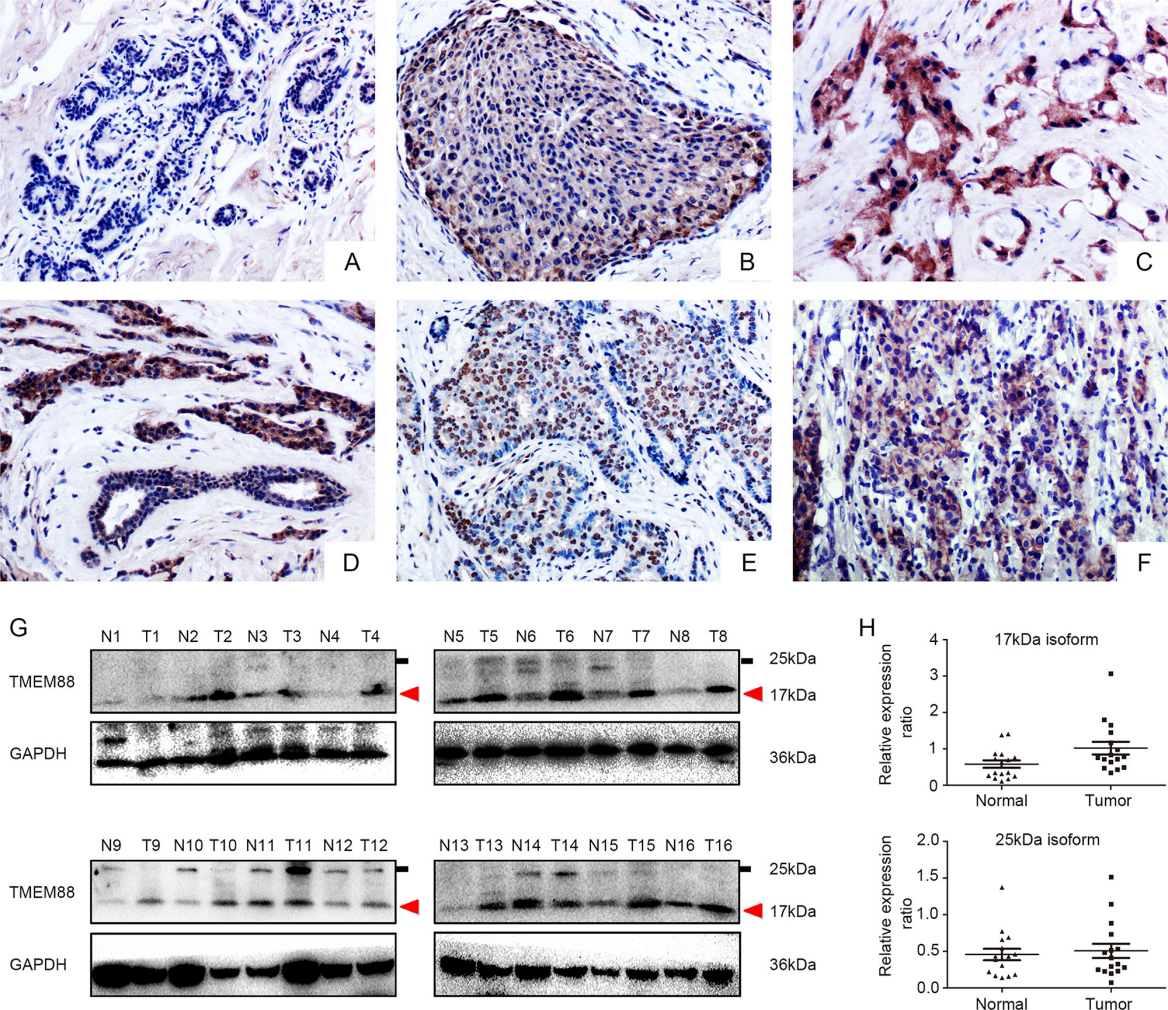
Expression and localization of target protein transmembrane 88(TMEM88) in breast cancer tissues **A.** TMEM88 expression was negative or low in normal breast ductal cells(200 ×), **B.** moderate in carcinoma tissues in situ(200×), **C.** and high in invasive ductal carcinomas(IDCs; 200×). **D.** TMEM88 expression was markedly higher in IDCs than in normal breast ductal cells (200×). **E.** TMEM88 was expressed at extremely high levels in the nuclei of certain samples (200×). **F.** Few tissue samples exhibited membrane localization of TMEM88 (200×). **G.** and **H.** Western blot analysis indicated significantly higher expression levels of the 17-kDa isoform of TMEM88 in the breast cancer tissues than in the paired noncancerous tissues. However, there was no significant difference in the expression levels of the 25-kDa isoform in breast cancer relative to the paired normal tissues.

We performed immunohistochemical analysis of 139 archived breast cancer specimens and their corresponding normal tissues. TMEM88 expression was negative or low (final score < 3; Figure 1A) in the normal tissues adjacent to the carcinomas. In contrast, TMEM88 was moderately expressed in carcinomas in situ and highly expressed in invasive ductal carcinomas (IDCs) (Figure 1B and 1C); however, the rate of positive expression was significantly higher in IDCs (71.22%, 99/139) than in normal breast ductal epithelium (11.4%, 4/35; *p* < 0.001; Figure 1D). Meanwhile, TMEM88 exhibited cytoplasmic and nuclear localization in 54.67% (76/139; Figure 1C) and 22.3% (31/139; Figure 1E) of the patient tissues, respectively. In contrast, plasma membrane localization was detected in only 2.1% (3/139; Figure 1F) of the cases. Moreover, the rates of positive cytosolic and nuclear expression in triple-negative breast cancers were 57.81% and 9.37%, while the corresponding rates in triple-positive breast cancer tissues were 52% and 33.33%, respectively (*p* = 0.5 and *p* = 0.001, respectively; Table 1). In both the overall breast cancer specimens and the triple-negative breast cancer specimens, cytosolic TMEM88 localization correlated positively with both lymph node metastasis (*p* = 0.01 and *p* = 0.002, respectively) and tumor node metastasis (TNM) stage (*p* = 0.038 and *p* < 0.001, respectively; Table 2). Meanwhile, nuclear localization of TMEM88 correlated negatively with lymph node metastasis (*p* = 0.046; Table 3), but was not associated with TNM stage in non-triple-negative breast cancers. In addition, nuclear localization was not associated with lymph node metastasis or TNM stage in the overall breast cancer specimens or in triple-negative breast cancers. These data indicate that cytosolic TMEM88 may promote tumor progression, while nuclear TMEM88 may inhibit this process.

**Table 1:** Nuclear expression of TMEM88 in triple-negative and triple-positive breast cancers

	ER, PR, Her2 (+)	ER, PR, Her2 (−)	
Cytosolic TMEM88	N		*p*
Positive	39	37	0.5
Negative	36	27	
Nuclear TMEM88			
Positive	25	6	0.001
Negative	50	58	

**Table 2:** Correlation of cytosolic TMEM88 overexpression with clinicopathologicalfeatures in breast cancer

Clinicopathological factors	All specimens			Triple-positive specimens			Triple-negative specimens		
	Positive	Negative	*p*	Positive	Negative	*p*	Positive	Negative	*p*
Age									
<52	33	36	0.126	20	24	0.241	13	12	0.604
≥52	43	27		19	12		24	15	
TNM classification									
I + II	49	51	0.038	34	27	0.239	15	24	<0.001
III	27	12		9	5		22	3	
Lymph node metastasis									
Positive	36	44	0.01	15	12	0.81	25	7	0.002
Negative	40	19		24	24		12	20	

**Table 3:** Correlation of nuclear TMEM88 overexpression with clinicopathologicalfeatures in breast cancer

Clinicopathological factors	All specimens			Triple-positive cancer			Triple-negative cancer		
	Positive	Negative	*p*	Positive	Negative	*p*	Positive	Negative	*p*
Age									
<52	20	49	0.116	16	28	0.322	4	21	0.199
≥52	11	59		9	22		2	37	
TNM classification									
I + II	25	75	0.263	23	38	0.122	5	34	0.391
III	6	33		2	12		1	24	
Lymph node metastasis									
Positive	9	50	0.102	20	28	0.046	4	28	0.672
Negative	22	58		5	22		2	30	

We next evaluated the expression levels of TMEM88 in 16 fresh breast cancer samples by western blot analysis. The normalized expression level of the 17-kDa TMEM88 isoform in breast cancer tissues (mean ± SD:1.019 ± 0.697) was significantly higher than that in the paired noncancerous tissues (mean ± SD:0.579 ± 0.408; *p* = 0.011; Figure 1G and 1H). However, there was no difference in the normalized levels of the 25-kDa TMEM88 isoform, which lacked the VWV sequence (Dvl-binding motif), in breast cancer tissues(mean ± SD: 0.506 ± 0.388) relative to the paired noncancerous tissues (mean ± SD: 0.457 ± 0.314; *p* = 0.472; Figure 1G and 1H). We therefore chose to include only the 17-kDa TMEM88 isoform (TMEM88 CRA-a; hereafter referred to as TMEM88) in subsequent analyses.

### Cytosolic TMEM88 interacts with Dvl to stimulate breast cancer cell invasion

**Figure 2:**
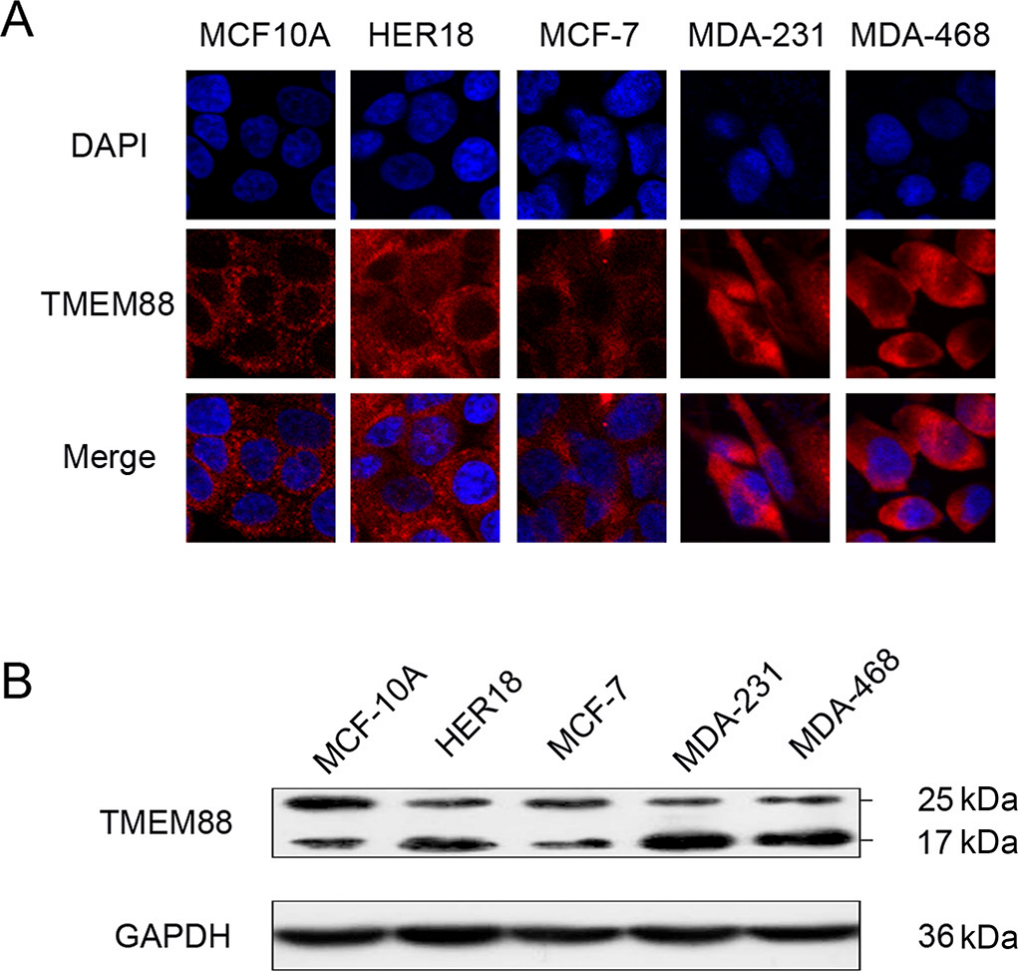
Expression and localization of target protein transmembrane 88 (TMEM88) in breast cancer cell lines **A.** Analysis of TMEM88 expression patterns in the MCF-10A, MCF-7, HER18, MDA-MB-231, and MDA-MB-468 cell lines by immunofluorescence microscopy using a TMEM88-specific antibody. Cells primarily exhibited cytoplasmic localization of TMEM88, but not plasma membrane or nuclear localization. **B.** In the majority of breast cancer cell lines, TMEM88 expression was higher than that in the normal breast cell line MCF-10A, and lower than that in the MCF-7 cell line. The 17-kDa TMEM88 isoform was expressed at significantly higher levels in the triple-negative breast cancer cell lines (MDA-MB-231 and MDA-MB-468) than in MCF-10A cells.

To further assess the expression level and pattern of TMEM88 protein in breast cancer tissues, immunofluorescence microscopy and western blotting analysis were utilized to TMEM88 expression in four breast cancer cell lines (MCF-7, HER18, MDA-MB-231, and MDA-MB-468). The normal breast cell line (MCF-10A) was used for comparison. While TMEM88 exhibited cytoplasmic localization in each of the cell lines tested (Figure 2A), the expression level of the 17-kDa TMEM88 isoform, which interacts with Dvl proteins, was significantly higher in the triple-negative breast cancer cell lines (MDA-MB-231 and MDA-MB-468) than in the normal breast cells (Figure 2B).

**Figure 3:**
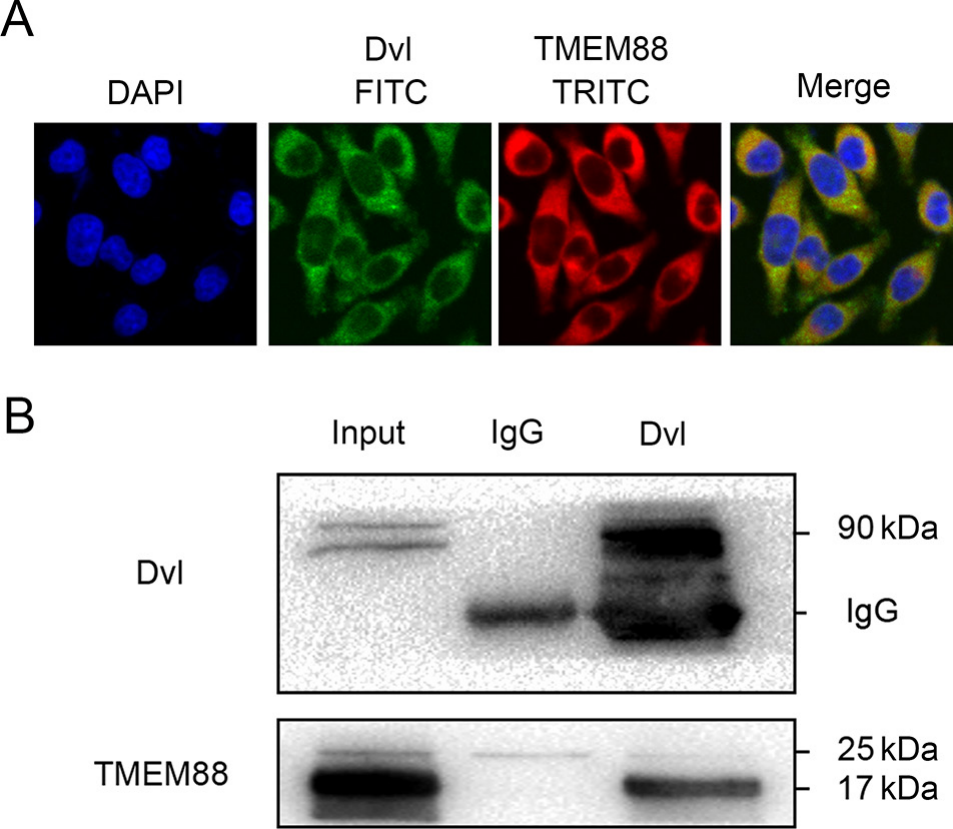
Target protein transmembrane 88(TMEM88) interacts with dishevelled (Dvl) **A.** Immunofluorescence staining analyses suggest that endogenous TMEM88 co-localized with Dvl in the cytoplasm of MDA-MB-231 cells. **B.** Co-immunoprecipitation analyses demonstrated that Dvl proteins interacted with TMEM88 in MDA-MB-231 cells, particularly with the 17-kDa isoform.

Immunofluorescence microscopy analyses using a Dvl-specific antibody indicated that TMEM88 co-localized with Dvl in the cytoplasm of the MDA-MB-231 cells (Figure 3A). Meanwhile, immunoprecipitation of Dvl resulted in pull-down of the 17-kDa isoform of TMEM88 (Figure 3B). These findings indicate that TMEM88 interacts with Dvl proteins in the cytoplasm of breast cancer cells.

**Figure 4:**
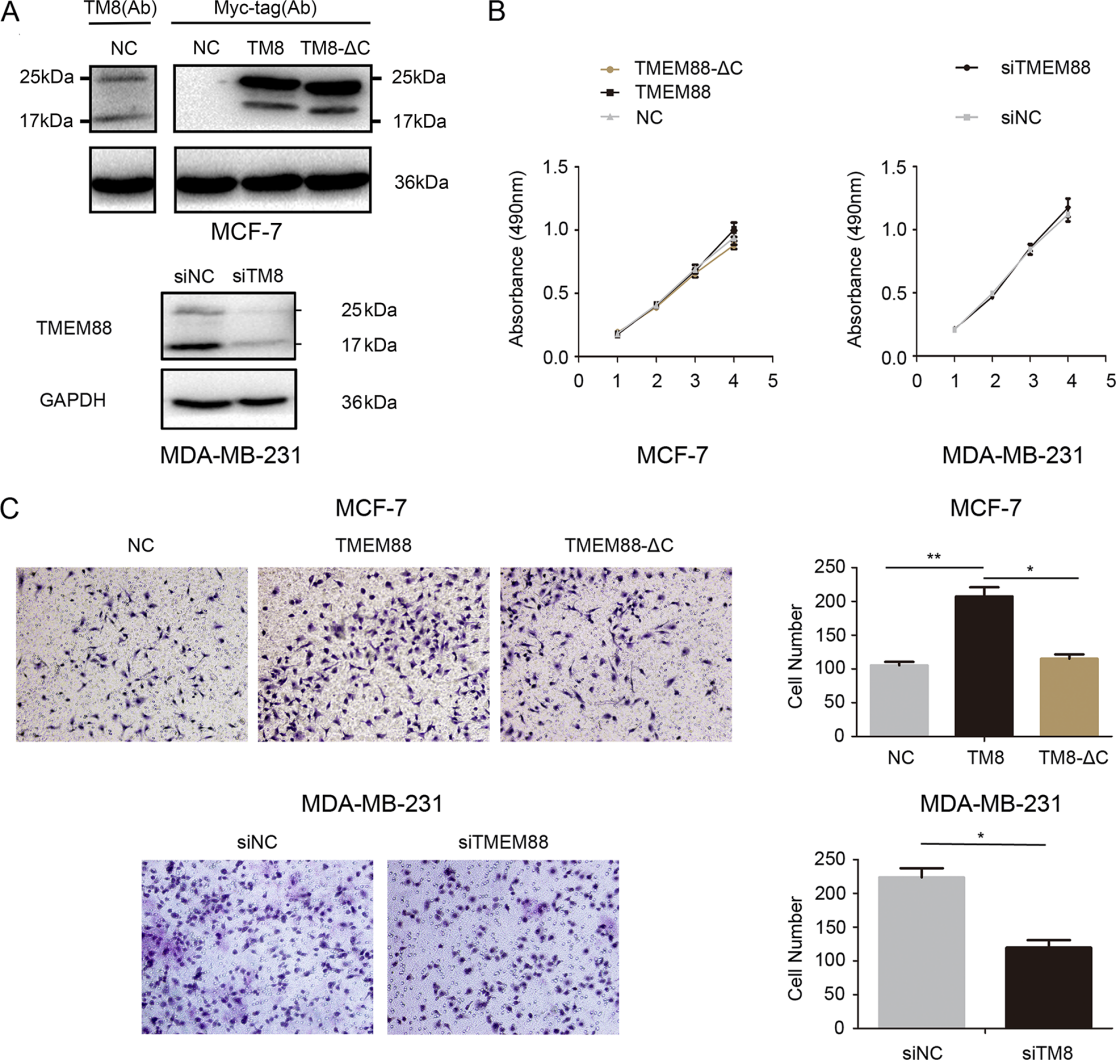
Effect of cytosolic target protein transmembrane 88 (TMEM88) on proliferation and invasion of breast cancer cells **A.** Western blot analyses were utilized to assess TMEM88 protein levels after overexpression or silencing of TMEM88 in MCF-7 and MDA-MB-231 cells, respectively. Two isoforms of endogenous TMEM88 (25 kDa and 17 kDa) were detected in MCF-7 cells using an anti-TMEM88 antibody. While a Myc-tag-specific antibody failed to detect the two endogenous isoforms of TMEME88 in cells overexpressing myc-TMEM88 (TMEM88 CRA-a), two exogenous bands that were greater than 17 kDa in size were observed. We propose that the band closest to 25 kDa in size may have resulted from targeting by two tag-specific (Myc and DKK) antibodies, while the other bands may comprise post-translationally modified versions of the protein. In cells expressing the TMEM88-ΔC variant, each of these bands was smaller in size, likely due to sequence truncation. **B.** MTT assay analyses detected no difference in the proliferation rates of MCF-7 cells overexpressing TMEM88 or TMEM88-ΔC and MDA-MB-231 cells transfected with the TMEM88-specific siRNA. **C.** Average number of migrating cells that passed through the pores (counted after 16 h). Treatment with the TMEM88 siRNA resulted in a drastic reduction in the invasion rate of MDA-MB-231 cells. Overexpression of TMEM88, but not TMEM88-ΔC, resulted in marked increases in the cell invasion rates of MCF-7 cells. Values represent means ± standard errors (bars) of three independent experiments; *p < 0.05, **p < 0.01 (Student's *t*-test); NC, negative control.

To examine the biological roles of cytosolic TMEM88 in breast cancer cells, we transfected MCF-7 with vectors expressing TMEM88 and TMEM88-ΔC (a TMEM88 variant that is unable to interact with Dvl). Furthermore, MDA-MB-231 cells were transfected with a control or TMEM88-specific siRNA to assess the effects of TMEM88 silencing in breast cancer cells (Figure 4A). MTT assay analyses indicated that there was no difference in cell proliferation between MCF-7 cells overexpressing TMEM88 and those overexpressing TMEM88-ΔC. Similarly, siRNA-mediated knockdown of TMEM88 expression did not significantly alter the proliferation of MDA-MB-231 cells (Figure 4B). Conversely, Matrigel invasion assays performed in Transwell plates showed that cell invasion significantly increased after TMEM88 overexpression, and markedly decreased after TMEM88 silencing. However, there were no obvious changes in cell invasion after transfection with TMEM88-ΔC. We made these observations. Our results indicate that cytosolic TMEM88 enhances breast cancer cell invasion, and that this process is dependent on the interaction between TMEM88 with Dvl proteins (Figure 4C).

### Cytosolic TMEM88 did not affect canonical Wnt signaling

**Figure 5:**
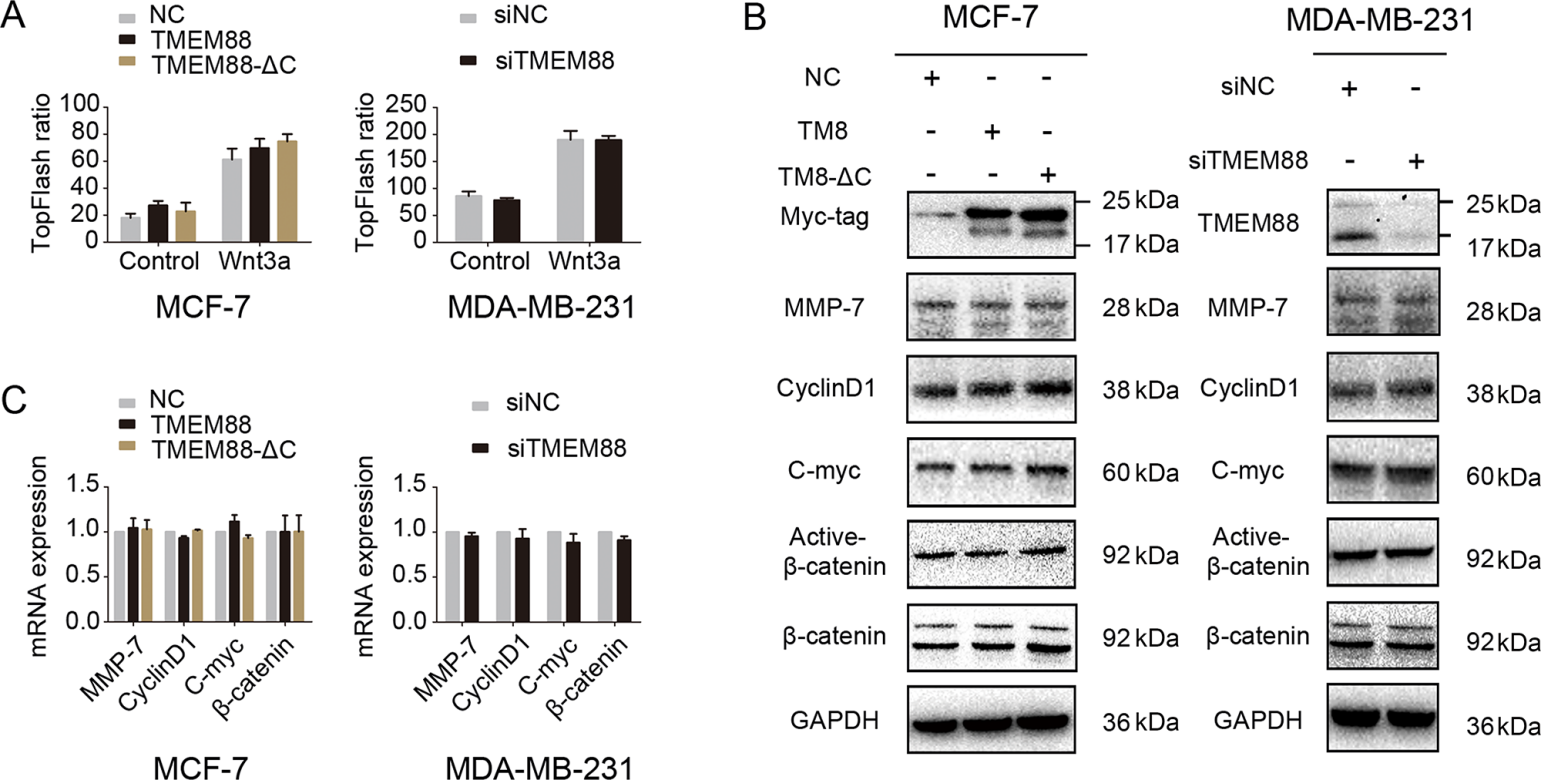
Cytosolic target protein transmembrane 88(TMEM88) did not affect canonical Wnt signaling **A.** Overexpression of TMEM88 and TMEM88-ΔC in MCF-7 cells and silencing of TMEM88 in MDA-MB-231 cells using a TMEM88-specific siRNA did not affect the activity of the TOPFlash reporter gene, regardless of treatment with Wnt3a. **B.** and **C.** Neither overexpression of TMEM88 and TMEM88-ΔC in MCF-7 cells nor silencing of TMEM88 in MDA-MB-231 cells affected the mRNA and protein expression levels of the target genes of the canonical Wnt signaling pathway (MMP-7, CyclinD1, and C-myc). Values represent means ± standard errors (bars) of three independent experiments; *p* > 0.05 (Student's *t*-test); NC, negative control.

To test whether TMEM88 stimulated cell invasion by activating the canonical Wnt signaling pathway, we performed luciferase assay, western blot, and real-time PCR analyses on MCF-7 cells overexpressing TMEM88 or TMEM88-ΔC and on MDA-MB-231 cells transfected with TMEM88-specific siRNAs. As shown in Figure 5A, dual luciferase assays indicated that neither the overexpression of TMEM88 or TMEM88-ΔC nor TMEM88 silencing had any effect on the activity of the canonical Wnt signaling pathway. These findings were supported by western blot and real-time PCR analyses, which detected no change the expression levels of the Wnt targets MMP-7, C-myc, and CyclinD1 upon overexpression of TMEM88 or TMEM88-ΔC or upon silencing of TMEM88 expression (Figure 5B and 5C). Together, these findings suggest that cytosolic TMEM88, independent of its interaction with Dvl, does not affect the activity of the canonical Wnt signaling pathway.

### TMEM88 inhibits occludin and Zo-1 by promoting snail expression

**Figure 6:**
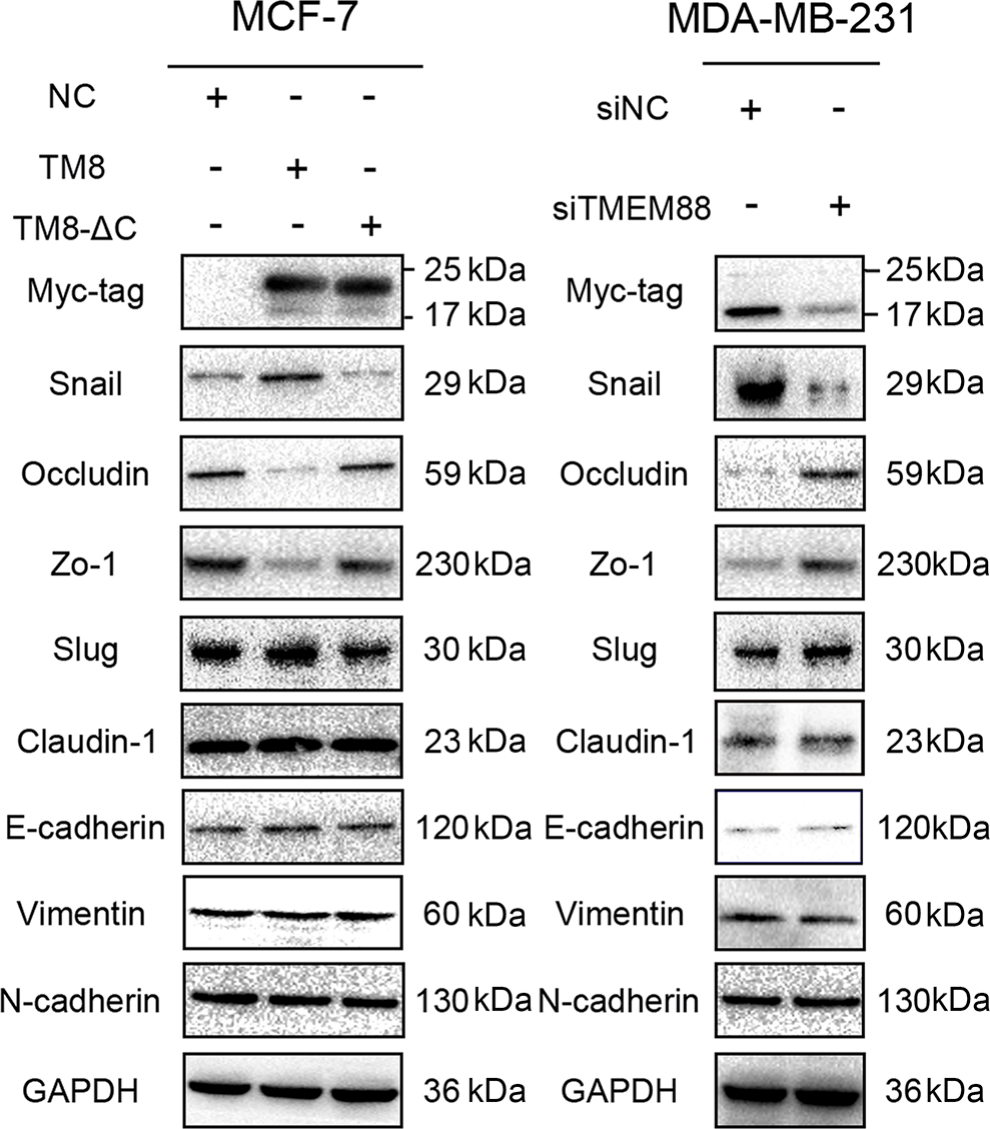
Target protein transmembrane 88(TMEM88) inhibits the expression of Occludin and Zo-1 by promoting Snail expression Overexpression of TMEM88 resulted in decreased expression of Occludin and Zo-1, and increased expression of Snail in MCF-7 cells; however, there were no significant changes in the expression levels of these proteins in cells overexpressing the TMEM88-ΔC variant. Meanwhile, contrasting results were observed in MDA-MB-231 cells transfected with the TMEM88-specific siRNA. However, modulation of TMEM88 expression had no effect on the expression levels of Claudin-1, E-cadherin, N-cadherin, and Vimentin in either cell line. At least three independent experimental replicates were performed and similar results were obtained for each experiment. Panels contain representative images.

We overexpressed TMEM88 or TMEM88-ΔC and silenced TMEM88 in MCF-7 and MDA-MB-231cells, respectively, to screen for the protein(s) involved in the epithelial–mesenchymal transition (EMT). Upon overexpression of TMEM88, there was an increase in Snail expression, and a concurrent decrease in Occludin and Zo-1 expression. Conversely, siRNA-mediated knockdown of TMEM88 expression resulted in reduced expression of Snail, and increased expression of Occludin and Zo-1. Meanwhile, overexpression of TMEM88-ΔC had no effect on Snail, Occludin, or Zo-1expression in MCF-7 cells (Figure 6). Likewise, modulation of TMEM88 expression had no effect on the expression levels of Claudin-1, E-cadherin, N-cadherin, and Vimentin. These findings indicate that TMEM88 promotes Snail expression in a Dvl protein-dependent manner.

## DISCUSSION

TMEM88 is a potential two-transmembrane–type protein that localizes to the cell membrane of *Xenopus* embryos [8]. In our study, only three specimens harvested from breast cancer patients exhibited membrane localization of TMEM88. In contrast, cytosolic TMEM88 expression was significantly higher in breast cancer tissues (54.67%, 76/139) than in carcinoma in situ specimens or in normal breast ductal tissues. Furthermore, cytosolic TMEM88 expression correlated with advanced TNM stage and lymph node metastasis. These results revealed that cytosolic TMEM88 might be associated with the malignant phenotype. To test whether the expression level of TMEM88 varied in breast cancers with distinct ER, PR, and Her2 expression patterns, the relationship between TMEM88 expression and clinicopathological factors was examined in triple-negative and triple-positive breast cancer tissues, respectively. There was no difference in the cytosolic expression rate between triple-negative and triple-positive breast cancers. However, cytosolic localization of TMEM88 was positively correlated with lymph node metastasis and TNM stage in triple-negative, but not triple-positive breast cancer tissues. These findings indicate that cytosolic localization of TMEM88 may be more closely associated with the malignant phenotype in triple-negative breast cancers, and could comprise a marker of poor prognosis in triple-negative breast cancer patients.

We also detected nuclear localization of TMEM88, and determined that this localization pattern primarily occurred in triple-positive breast cancer tissues, where it negatively correlated with lymph node metastasis. As such, nuclear localization of TMEM88 could be considered an indicator for positive prognoses in triple-positive breast cancer patients. Thus, it appears that the differential localization patterns of TMEM88 are associated with distinct prognoses. However, the biological role of nuclear localization of TMEM88 in triple-positive breast cancer tissues, as well as the mechanism by which this protein translocates into the nuclei of these cells are currently unclear. These points, in addition to the relationship between nuclear TMEM88 localization and ER, PR and Her2 expression, therefore require further study. Indeed, characterization of the biological roles of the distinct subcellular localization patterns of TMEM88 may provide new insights into targeted therapy for breast cancer.

Lee et al. (2010) reported that TMEM88 interacts with Dvl by competing with LRP5/6, leading to recruitment of Dvl to the plasma membrane and subsequent inhibition of the canonical Wnt signaling pathway. However, our results indicate that TMEM88 localizes primarily to the cytoplasm and that overexpression of this protein enhances cell invasion. Meanwhile, co-immunoprecipitation and immunofluorescence analyses demonstrated that TMEM88 interacts with Dvl within the cytoplasm of cancer cells. Furthermore, our results indicate that cytosolic TMEM88, independent of its interaction with Dvl, does not affect the activity of the TopFlash reporter or the expression levels of the target genes of the canonical Wnt signaling pathway. We therefore propose that the differential effects on canonical Wnt signaling may be attributable to the distinct subcellular localization patterns of TMEM88.

We explored the potential mechanism by which cytosolic TMEM88 stimulates breast cancer cell invasion by examining the expression levels of the proteins involved in the EMT. We detected enhanced expression of Snail, and reduced expression of Occludin and Zo-1 in cells transiently expressing TMEM88. In contrast, the expression levels of Snail, Occludin, and Zo-1 were unaltered in cells overexpressing the TMEM88-ΔC variant. These results suggest that the observed TMEM88-mediated modulation of the expression levels of these proteins occurred in a Dvl-dependent manner. However, the mechanism by which the interaction between TMEM88 and Dvl promotes the expression of Snail is still unclear. Previous studies demonstrated that the Wnt, transforming growth factor β (TGFβ), mitogen-activated protein kinase (MAPK), and phosphatidylinositol 3-kinase (PI3K)/Akt signaling pathways are involved in the upregulation of Snail expression [10–17]. Furthermore, glycogen synthase kinase-3 β (GSK3β) and nuclear factor (NF)-κB were found to be the key regulating proteins that govern this process [18–23]. Meanwhile, it was also reported that overexpression of Dvl can result in p38 and JNK MAPK activation (i.e., phosphorylation) [24, 25]. However, future studies are necessary to determine whether the TMEM88/Dvl-mediated modulation of Snail, Occludin, and Zo-1 expression occurs via activation of the p38 or JNK signaling pathways.

In summary, TMEM88 was highly expressed in both breast cancer tissues and cell lines and primarily exhibited cytoplasmic localization in these tissues. Furthermore, the cytosolic expression of this protein was positively correlated with lymph node metastasis and TNM stage in triple-negative breast cancer. We demonstrated that cytosolic TMEM88 interacted with Dvl in breast cancer cell lines, while this interaction resulted in increased Snail expression, decreased expression of Occludin and Zo-1, and stimulation of breast cancer cell invasion, it did not affect the activity of the canonical Wnt signaling pathway. Notably, TMEM88 was primarily localized within the cell nuclei of triple-positive breast cancer tissues, and this nuclear localization pattern was negatively correlated with lymph node metastasis. Indeed, the expression patterns of TMEM88 were noticeably different in triple-negative compared to triple-positive breast cancers. We propose that these variable expression patterns may be associated with divergent biological roles that occur via distinct mechanisms.

## MATERIALS AND METHODS

The study protocol was approved by the institutional review board of China Medical University. All participants provided written informed consent, and the study was conducted according to the principles expressed in the Declaration of Helsinki.

### Patients and specimens

Primary tumor specimens were obtained from 139 patients (64 cases of triple-negative and 75 cases of triple-positive breast cancer) who were diagnosed with IDC and underwent complete surgical resection at the First Affiliated Hospital of China Medical University between 2008 and 2013. None of the patients had received radiotherapy or chemotherapy before undergoing surgical resection, and all patients received routine chemotherapy after surgery.

For comparison with the immunohistochemical data, fresh tumor and paired noncancerous tissues were collected from 16 patients and immediately stored at −70°C. Samples were then subjected to protein extraction and western blot analysis.

### Cell lines

The MCF-10A, MCF-7, HER18, MDA-MB-231, and MDA-MB-468 cell lines were obtained from Shanghai Cell Bank (Shanghai, China). All cells were cultured in RPMI 1640 medium (Invitrogen, Waltham, MA, USA) containing 10% fetal calf serum (Invitrogen), 100 IU/mL penicillin, and 100 μg/mL streptomycin (Sigma-Aldrich, St. Louis, MO, USA). The cells were grown in sterile culture dishes at 37°C with 5% CO_2_, and passaged every 2 days using 0.25% trypsin (Invitrogen).

### Immunohistochemical analysis

All tissue specimens were fixed in neutral formaldehyde, embedded in paraffin, and sectioned to a thickness of 4 μm. The streptavidin-peroxidase immunohistochemical method was used to improve the staining. Briefly, tissue sections were incubated at 4°C overnight with TMEM88-specific rabbit polyclonal antibody (1:50 dilution; Sigma). As a blank control, phosphate-buffered saline was used in place of the antibody. The sections were then incubated with biotin-labeled secondary antibodies (Ultrasensitive; Fuzhou MaiXin Biotechnology Development Co., Ltd., Fujian, China) at 37°C for 30 min, and then with diaminobenzidine for coloration.

The intensity of TMEM88 staining was scored as follows: 0, no signal; 1, weak; 2, moderate; and 3, high. Percentage scores were assigned as follows: 1, 1%–25%; 2, 26%–50%; 3, 51%–75%; and 4, 76%–100%. The scores of each tumor sample were multiplied to give a final score of 0–12. For analysis of cytosolic TMEM88 localization, tumors with final scores ≥3 were characterized as exhibiting cytosolic overexpression of TMEM88, while those with final scores <3 were characterized as having negative or weak cytosolic TMEM88 expression. Meanwhile, positive nuclear TMEM88 localization was indicated by nuclear staining scores >2 and percentage scores >5%. In cases where TMEM88 was localized in both the cytoplasm and nucleus, only the nuclear expression was counted.

### Western blot and immunoprecipitation analyses

Total cellular protein was extracted from tissues using lysis buffer (Pierce Biotechnology, Waltham, MA, USA) and quantified using the Bradford method. Approximately 50 μg of each protein sample was separated by 10% sodium dodecyl sulfate polyacrylamide gel electrophoresis (SDS-PAGE), transferred to polyvinylidene fluoride (PVDF) membranes (Millipore, Billerica, MA, USA), and incubated with primary antibodies in blocking buffer overnight at 4°C. TMEM88- and GAPDH-specific antibodies (diluted 1:500 and 1:5000, respectively) were purchased from Sigma; cyclinD1- and Dvl-specific antibodies(1:100 for each) were purchased from Santa Cruz Biotechnology Inc. (Dallas, TX, USA);antibodies against Snail, Slug, matrix metalloproteinase (MMP)-7, Myc-tag, Vimentin, and active β-catenin (1:1000 for each) were purchased from Cell Signaling Technology (Danvers, MA, USA); antibodies specific to β-catenin, E-cadherin, N-cadherin, and C-myc (1:1000 for each) were purchased from BD Transduction Laboratories (BD Biosciences; San Jose, CA, USA); the claudin-1(1:2000) antibody was purchased from Invitrogen; and antibodies specific to Zo-1 and Occludin (1:500) were purchased from Proteintech(Chicago, IL, USA).

For immunoprecipitation experiments, a sufficient amount of antibody was added to 200 mg of protein and gently rotated overnight at 4°C. Immunocomplexes were captured by adding 25 μL of protein A/G agarose beads (Beyotime, Beijing, China) and gently rotating for 3 h at 4°C. The mixtures were then centrifuged at 1500 × *g* for 5 min at 4°C, and the supernatants were discarded. Precipitates were washed three times with ice-cold radioimmunoprecipitation assay (RIPA) buffer, resuspended in sample buffer, and boiled for 5 min to dissociate the immunocomplex from the beads. Supernatants were then collected by centrifugation and subjected to western blot analysis.

### Plasmid construction and transfection

The pCMV6-DDK-Myc empty vector and the pCMV6-DDK-Myc-TMEM88 CRA-a (TMEM88) vector were purchased from OriGene (Rockville, MD, USA), the TMEM88-ΔC vector was constructed by Takara Bio Inc. (Dalian, China), and the Super8 × TOPFlash, Super8 × FOPFlash, and pRL-TK vectors were purchased from Addgene (Cambridge, MA, USA). The TMEM88-siRNA (sc-93891) and NC-siRNA (sc-37007) were purchased from Santa Cruz Biotechnology. Transfection was carried out using a Lipofectamine 2000 kit (Invitrogen), according to the manufacturer's instructions.

### Immunofluorescencestaining

Cells were fixed with 4% paraformaldehyde, blocked with 1% bovine serum albumin, and incubated overnight with the TMEM88- (1:100; Sigma) and Dvl-specific polyclonal antibody antibodies (1:100, Santa Cruz Biotechnology) at 4°C. The following morning, the cells were incubated with tetramethylrhodamineisothiocyanate-conjugated secondary antibodies at 37°C for 2 h. The nuclei were counterstained with 4′, 6-diamidino-2-phenylindole (DAPI). Epifluorescence microscopy was performed using an inverted Nikon TE300 microscope (Nikon Co., Ltd., Tokyo, Japan), and confocal microscopy was performed using a Radiance 2000 laser scanning confocal microscope (Carl Zeiss, Oberkochen, Germany).

### Dual-luciferase assay

Cells were seeded in 24-well plates, incubated for 24 h at 37°C, and then transfected with the control plasmid pRL-TK (50 ng) and either the TOPFlash or FOPFlash (0.5 mg) plasmids using lipofectamine 2000 reagent, according to the manufacturer's instructions. After incubation for 30 h at 37°C, reporter gene expression was detected using the Dual-Luciferase Assay System (Promega, Madison, WI, USA). Recombinant human Wnt3a (R&D Systems, Minneapolis, MN, USA) was reconstituted at 10 μg/mL in PBS containing 0.1% BSA, and used in experiments at a final concentration of 100ng/mL. Tcf-mediated gene transcription was determined by measuring the ratio of TOPFlash to FOPFlash luciferase activity and normalizing to the *Renilla* luciferase activity generated from the pRL-TK control plasmid. All experiments included two replicates and were repeated a minimum of three times. To analyze the effect of TMEM88 on β-catenin signaling, the TMEM88 expression vector (0.5 mg) was co-transfected with TOPFlash or FOPFlash, and luciferase activity was assessed. Where necessary, empty vectors (0.5 mg) were added to transcription reactions to control for the amount of plasmid DNA.

### RNA extraction and quantitative real-time reverse transcription polymerase chain reaction (qRT-PCR) analysis

Total cellular RNA was extracted from cells using an RNeasy Plus Mini Kit (Qiagen, Venlo, Netherlands). qRT-PCR was performed using SYBR Green PCR Master Mix (Applied Biosystems, Carlsbad, CA, USA), and reactions were carried out in 20 μL volumes. Samples were analyzed using a 7900 Real-Time PCR System (Applied Biosystems) and the following amplification parameters: 50°C for 2 min and 95°C for 10 min, followed by 40 cycles of 95°C for 15 s and 60°C for 60 s. The sequences of the primer pairs used in this study are listed in Table 4; β-actin expression levels were used as a reference. The relative levels of gene expression were represented as ΔCt (cycle threshold) = Ct gene/Ct reference, and fold changesin gene expression were calculated using the 2^−ΔΔCt^method. All experiments were repeated in triplicate and three experimental replicates were performed for each sample per experiment.

**Table 4:** Primers used in this study

	Primer sequences (5′→3′)
MMP-7	Forward 5′-TCGGAGGAGATGCTCACTTCGA-3′ Reverse 5′-GGATCAGAGGAATGTCCCATACC-3′
CyclinD1	Forward 5′-TGGAGGTCTGCGAGGAACA-3′ Reverse 5′-TTCATCTTAGAGGCCACGAACA-3′
C-myc	Forward 5′-GCCACGTCTCCACACATCAG-3′ Reverse 5′-TGGTGCATTTTCGGTTGTTG-3′
β-catenin	Forward 5′-CACAAGCAGAGTGCTGAAGGTG-3′ Reverse 5′-ATAGCACAGCCTGGATAGCAACGTAC-3′
β-actin	Forward 5′-ATAGCACAGCCTGGATAGCAACGTAC-3′ Reverse 5′-CACCTTCTACAATGAGCT GCGTGTG-3′

### MTT assay

Cells transfected with a TMEM88 expression vector or with either the control or TMEM-specific siRNA were seeded at a concentration of 3,000 cells per well in 96-well plates containing medium supplemented with 10% fetal bovine serum (FBS). For quantitation of cell viability, cultures were incubated for 4 days at 37°C with 5% CO_2_ and subjected to MTT assay analysis. In brief, 20 μL of 5 mg/mL MTT (3-(4, 5-dimethylthiazol-2-yl)-2, 5-diphenyltetrazolium bromide; thiazolyl blue) solution was added to each well and incubated for 4 h at 37°C. The medium was then removed from each well, and the resultant MTT formazan was solubilized in150μL dimethyl sulfoxide. The results were quantitated spectrophotometrically using a test wavelength of 490 nm.

### Matrigelinvasion assay

Cell invasion assays were performed using 24-well Transwell chambers with a pore size of 8 μm (Costar, Corning Life Sciences, Tewksbury, MA, USA); the inserts were coated with 20 μL Matrigel (1:3 dilution; BD Biosciences). MCF-7 and MDA-MB-231 cells were transfected with TMEM88 expression vectors or with control or TMEM88-specific siRNAs, respectively, incubated for 48 h at 37°C with 5% CO_2_, harvested by trypsin digestion, diluted to a concentration of 3 × 10^5^ cells in 100 μL serum-free medium, transferred to the Matrigel-treated chambers of Transwell plates, and incubated for 16 h at 37°C with 5% CO_2_. Medium supplemented with 10% FBS was added to the lower chamber and utilized as the chemoattractant. The non-invading cells on the upper membrane surface were removed with a cotton tip, while the cells that had passed through the filter were fixed with 4% paraformaldehyde and stained with hematoxylin. The invading cells in 10 randomly selected high-power fields were counted under a microscope. Experiments were performed in triplicate.

### Statistical analyses

All statistical analyses were performed using SPSS 17.0 (SPSS Statistics, Inc., Chicago, IL, USA) software. The immunohistochemistry results were analyzed by chi-square and Spearman's rank correlation analyses. Differences between groups were compared using Student *t*-tests; *p* < 0.05 was considered statistically significant.
